# Antiseptics in Ophthalmology

**Published:** 1880-10

**Authors:** Lucien Howe


### ANTISEPTICS IN OPHTHALMOLOGY.

FROM THE SPANISH BY LUCIEN HOWE, M. D.

THE eminent Professor Wecker, of Paris, while on a visit to Madrid recently, delivered a lecture before the leading men in the medical profession of that city on this important subject. The following extracts are taken from the report in the *Cronica Oftalmologica* ano X num I.

* * * "Without further delay, then, I proceed at once to the topic, the subject of my discourse being antiseptic therapeutics considered in an ophthalmological point of view, and as connected thus with medicine as a whole. Let us, therefore, study some of the diseases of the eye, which are related to general medicine, commencing with those which affect the conjunctiva, especially purulent ophthalmia of children or of adults.

"As regards this trouble in the former class, I believe it will be avoided in the future by causing such mothers as suffer from an evident gonorrhea, to make use of disinfecting injections during the time of pregnancy. Among adults, the development of the disease is always preceded by a local infection from persons having the disease. * * *

"I would not say it was necessary that this infection should proceed directly from the individual. It frequently happens in eastern countries, that isolated persons contract a purulent ophthalmia without coming in contact with others, beginning thus as a simple catarrhal affection induced by climatic influences. But as the conjunctival secretion accumulates along the edges of the lids, and as these people are not given to frequent ablutions, putrefactive changes are produced by the warmth, and a purulent ophthalmia thus established. In such an instance, the individual poisons himself, and the more one studies the subject the more evident does it become that in all cases there is infection. What is more natural then, than infection should be treated by disinfection? * * * *

"I would also call your attention to the treatment of wounds of the cornea and particularly, to what occurs in the capital operation for cataract. By a careful study of these conditions one is convinced, even when the lesions are of traumatic character, that they are subject to infection and are to be considered all the more threatening in proportion, as this is intense. It is impossible not to think that all suppurations of the cornea, which follow operations, are due to infection. I consider it criminal to operate on an eye having already a contagious trouble, such, for example, as occurs in the lachrymal passage, in the conjunctiva or along the borders of the lids, nor should we operate for cataract without previously disinfecting the eye, whether we know it has been exposed or not.

"With some few modifications, Lister's method, with all its details, can be used in ophthalmic practice. I did believe, and I have said in my *Therapie Oculaire*, that the use of the atomizer was irritating to the eye, but during the past year I have been convinced from constant trial, that I was wrong. Carbolic acid spray, as employed by Lister, produces no irritation, nor does it hinder the operation for cataract, nor retard the cure. I should also say a few words concerning the best disinfectants for use in diseases of the eye.

"Carbolic acid solutions, about two parts to one hundred, have the inconvenience of smarting somewhat, and irritating the lids;

Salicylic acid is faulty in being easily precipitated from its solutions, and deposits crystals upon the cornea, as has been shown by experiments practiced upon animals; and although this has not been observed in the human subject, still the objection is worthy of consideration. Boracic acid has the evident advantage of being free from odor and perfectly harmless, but like the last, is not an active disinfectant unless used in very concentrated solutions. By mixing the last two substances, however, a better and more stable solution is obtained.

" For this purpose, especially in corneal troubles, and for disinfection of the eye as a whole, I make use of a formula containing twenty parts of boracic acid, with two of salicylic acid to one hundred of water.*

"And while speaking of corneal diseases, I should also mention agents which are in vogue for their treatment, especially the sulphate, or better, the salicilate of esserin and the sulphate and muriate of quinine.

" Esserin is very frequently employed in corneal affections, and is, to a certain extent, I think, well worthy of the reputation it has won. It has been clearly shown, however, that this is not a disinfectant. What has it then to do with a lecture on antiseptics? The infection of a corneal wound inevitably produces suppuration, which is itself immediately caused by a wandering of the white blood corpuscle through the minute openings—stomata—of the blood vessels.

" Now, experiments upon animals have shown that esserin contracts these openings in the walls of the vessels, and is therefore an active agent in controlling such transudations. You see, then, how this can retard the suppurative effect of infection without being strictly an antiseptic, and thus can be explained the importance which has been attributed to it in corneal affections, although not acting as a disinfectant.

* There must be some mistake in the proportions as here given. Boracic acid is not so readily soluble in cold water. "Crystallized boracic acid is insoluble in ether, soluble in alcohol, and requires about 30 parts of cold, and 3 parts of boiling water for solution."—*Nat. Dispensatory.*—Tr.

"According to recent experiments, verified at Rome and other points in Italy, we now know that in the atmosphere of those districts, where low forms of intermittent (paludine) fevers are endemic, their exist real organisms, which, being absorbed, affect individuals; unquestionably we may impute to these substances, the more or less pernicious effect of such fevers. If it should be doubted that the first febrile disturbance of a purulent infection (which might be produced by the absorption of matter from an ulcerating cornea), should be treated by the exhibition of muriate of quinine, we must at least admit, that we treat the conditions resulting in these two diseases—namely, intermittent fever and purulent infection—by the internal use of sulphate of quinine. It would, therefore, appear that this agent should be classed among the more important of our antiseptics.

"Then leaving this special department, let us pass over to the more extended field of general medicine, although internal affections of the eye, can not be produced by direct contagion on account of the impenetrable membranes enveloping it.

"Let us take a variety of iritis for example. Suppose a man has a gonorrhœa, and from negligence or from excessively strong injections, there results an articular rheumatism, and gonorrhœal iritis, what relation is there between these three diseases? You may know, gentlemen, that experiments recently made in Germany, with special reference to acute articular rheumatism, have shown, that in the blood of those suffering from it, there exist certain organisms analogous to those found with paludine fevers and in septicæmia. We notice, also, that attacks of rheumatism come sometimes like a flash, the individual being seized with a high fever—attacks which I think have been quite aptly regarded as cerebral rheumatism.

"I call attention thus to the relation between gonorrheal rheumatism and gonorrhœal iritis, and between chronic rheumatism and rheumatic iritis, in order to show how it happens, as to the latter disease, (iritis) that the infecting elements may pass into the blood from a gonorrheal secretion—this having entered the circulation through some abraded spot of mucous membrane—and thus produce a gonorrheal rheumatism or iritis.

"In my *Therapie Oculaire* it is asserted that "this should be considered a question of physics," and an eminent surgeon of Munich, Professor Nussbaum, without knowing of that declaration, has repeated it. I have said before that therapeutics must be rational to be of any value, and I would now say that there must also be an antiseptic therapeusis, or there can be no other."

